# Antegrade Continence Enema vs. Botulinum Toxin in Pediatric Chronic Idiopathic Constipation: A 10-Year Retrospective Study at a Single Center

**DOI:** 10.3390/children12111565

**Published:** 2025-11-18

**Authors:** Prisca C. Obidike, Trevor C. Jones, Chioma Moneme, Alexander Bills, Zoë Hemmer, Alison Jung, Lillian Wu, Lily S. Cheng

**Affiliations:** Department of Surgery, University of Virginia, Charlottesville, VA 22903, USA; hyf2gr@uvahealth.org (P.C.O.);

**Keywords:** chronic idiopathic constipation, antegrade continence enema, botulinum toxin, functional constipation, encopresis

## Abstract

**Highlights:**

**What are the main findings?**

• Antegrade continence enemas (ACE) and Botulinum Toxin (BT) both improved oral laxative use and constipation-related symptoms at one year postoperatively from baseline.
• ACE recipients had a greater reduction in oral laxative use, while BT was associated with fewer complications and healthcare utilization visits at 1 year postoperatively.

**What is the implication of the main finding?**

• Decision-making for surgical interventions in pediatric CIC should be individualized and balanced between effectiveness and healthcare utilization.

**Abstract:**

Introduction: Chronic Idiopathic Constipation (CIC) is a common pediatric gastrointestinal disorder (GI) characterized by persistent difficulty in defecation, with no identifiable underlying cause. Although most patients are successfully treated with medical therapies, surgical intervention is often needed for refractory disease. We evaluated the impact of Antegrade Continence Enemas (ACE) and Botulinum Toxin (BT) injection to the internal anal sphincter on laxative use, symptom resolution, and healthcare utilization. Methods: A retrospective chart review was conducted to identify patients ≤ 18 years old presenting to a pediatric surgery clinic with a chief complaint of CIC between 1 March 2014 and 1 March 2024. Patients meeting the Rome IV criteria for idiopathic constipation and fecal incontinence were included. Surgical procedures were categorized into BT injection or ACE channel creation. The primary outcome was change in daily oral laxative use at 1 year, and secondary outcomes included symptom resolution and CIC-healthcare utilization at 1 year postoperatively. Results: Of the 125 children who presented with CIC, 47 (37.6%) underwent surgery. Mean age was 6 years at the time of surgery. 17 (36.2%) had ACE channel creation, while 30 (63.8%) received BT injections. At 1 year, daily oral laxative polypharmacy decreased from 60.2% to 41.0%, *p* < 0.001, with a greater reduction in ACE than BT (adjusted mean difference: −1.05, 95% CI: −1.75 to 0.34, *p* = 0.004) after adjusting for demographics and baseline clinical factors. Overall, symptom resolution of encopresis (79.1% to 39.5%, *p* = 0.001), abdominal pain (88.4% to 27.9%, *p* < 0.001), and abdominal distension (67.4% to 27.9%, *p* < 0.001) was observed with no significant difference between groups at 1 year. ACE patients had significantly more postoperative outpatient CIC-related visits and no change in ED visits compared to fewer visits in BT patients. Conclusions: Both ACE and BT recipients had improvements in constipation-related symptoms and laxative use. However, ACE resulted in a significantly greater reduction in daily laxative use and more postoperative CIC-healthcare visits than BT alone.

## 1. Introduction

Chronic constipation is one of the most common gastrointestinal disorders, affecting up to 30% of children and adolescents [1,2,3]. It is defined as infrequent or difficult stool passage, or a sensation of incomplete evacuation, persisting for at least three months [2,4,5]. Although structural, inflammatory, and metabolic abnormalities can cause constipation, the majority of pediatric cases lack an identifiable etiology and are classified as chronic idiopathic constipation (CIC) [6,7]. CIC significantly impairs quality of life and incurs a substantial economic burden on the healthcare system [8,9,10,11,12,13]. In the United States alone, CIC-related encounters account for more than 2.5 million hospital and physical visits per year, resulting in $3.9 billion in annual healthcare costs. First-line management includes medical and behavioral therapies such as laxatives, enemas, and dietary modification. However, children with moderate to severe forms of CIC, particularly those with encopresis or soiling, intractable abdominal pain, distension, or fecal impaction, often remain refractory to medical therapy and may require surgical intervention [7,14].

Surgical management of pediatric constipation includes anal procedures, antegrade continence enemas (ACE), and, in refractory cases, intestinal diversion or resection [7,15,16]. Among anal procedures, botulinum toxin (BT) injection into the internal anal sphincter (IAS) is preferred over myotomy, myectomy, or anal dilatation due to its minimally invasive nature, safety, and effectiveness [15,17]. ACE procedures, including Malone appendicostomy and cecostomy, provide direct colonic access for irrigation and are effective in improving symptoms, though complications such as infection, leakage, or stenosis may require revision or reversal [18,19]. More invasive options, including diversion (ileostomy or colostomy) or colorectal resection, are typically reserved for the most severe and refractory cases [15]. No single method has been established as the best practice for CIC, and surgical approach often varies by patient and provider.

BT injections and ACE channels are common surgical treatments for medically refractory CIC; however, there is limited comparative data on their outcomes [15,16,17,20]. To date, no studies have directly compared ACE and BT in children, and there are no guidelines to inform treatment selection. We conducted a retrospective cohort study of pediatric patients with CIC who had surgery at a single institution over a 10-year period to compare the effectiveness of BT and ACE in clinically important treatment outcomes: symptom resolution, change in laxative use, and healthcare utilization. Our findings demonstrate the effectiveness of ACE and BT in symptom resolution and oral laxative use reduction. Despite a greater reduction in laxative use in ACE recipients, healthcare utilization and complication rates were higher at one year postoperatively.

## 2. Materials and Methods

With permission from our institutional review board (IRB), we performed a retrospective review of patients presenting with a chief complaint of chronic idiopathic constipation (International Classification of Diseases, 10th revision code: K59.04) to the pediatric surgery clinic at the University of Virginia between 1 March 2014 and 1 March 2024. Informed consent was not required for this study. The study was conducted in accordance with the Declaration of Helsinki, and the protocol was approved by the IRB of the University of Virginia (HSR230261) on 7 September 2023. Inclusion criteria included patients ages 0–18 years with symptoms consistent with chronic idiopathic constipation according to the Rome IV criteria [21,22], lasting at least three months. Exclusion criteria included Hirschsprung disease, anorectal malformations, congenital colorectal disease, spina bifida, inflammatory bowel disease, metabolic abnormalities, and other known causes of constipation. Patients with other comorbidities or history of prior abdominal surgery not directly related to constipation or the outcomes of interest were not excluded. Among eligible patients, those who underwent surgical intervention, specifically ACE channel placement or BT injection, were identified and categorized by procedure type. The ACE group included patients who underwent Malone appendicostomy or cecostomy button placement as previously described in the literature [23,24]. The BT group comprised patients who received 100 units of BT injected into the IAS, as previously described [25]. Patients who underwent ACE channel placement after BT injection were categorized as the ACE group. Patients who received multiple BT injections were analyzed based on their response to their first BT injection.

Data collected included demographics (age, sex, race/ethnicity), insurance provider, home-to-hospital travel distance, and Area Deprivation Index (ADI) [26,27] percentile as a measure of socioeconomic status. Clinical characteristics, such as diagnostic testing for constipation, constipation-related symptoms, and the presence of a neurodiverse disorder were also obtained. Neurodiverse disorders were defined as autism spectrum disorder (ASD), anxiety, attention-deficit/hyperactivity disorder (ADHD), or intellectual and developmental disabilities (IDD). The authors included neurodiverse disorders given the well-documented association between neurodiversity and constipation [28,29,30,31]. Constipation-related variables assessed included abdominal pain, abdominal distension, bowel movement frequency, encopresis, and oral laxative use. Bowel movement frequency was reported as the number of bowel movements per week (BM/wk). Oral laxatives were categorized by mechanism (osmotics, stimulants, secretagogues) and total number of laxative agents taken daily (e.g., polyethylene glycol, senna, milk of magnesia).

Among patients who underwent surgical intervention, including both ACE and BT, data collected included age at surgery, frequency of GI and constipation-related healthcare visits per year before and one year after surgery, and 30-day postoperative complications. The primary outcome was the change in the total number of daily oral laxative agents used from baseline to one year postoperatively. Secondary outcomes included resolution of abdominal pain, distension, and encopresis, if present. The frequency of BM/wk and changes in GI-related healthcare visits were also examined over the same period. These data were abstracted from patients’ electronic medical records including doctors’ notes from GI-related healthcare visits. Patients who were lost to follow-up were excluded from the analysis of outcomes at the corresponding evaluation time points.

Numeric variables were summarized as mean with standard deviation (SD). Categorical variables were reported as frequency and percentage. Between-group comparisons were performed using McNemar for paired and Chi-square or Fisher’s exact tests for unpaired categorical variables. A paired Student’s *t*-test was used for continuous variables. Univariate and multivariate linear regression analyses were used to evaluate the outcomes of interest while accounting for potential confounders. Statistical analyses were conducted using R Studio (version 2024.04.2+764; Boston, MA, USA). A *p*-value < 0.05 was considered statistically significant.

## 3. Results

### 3.1. Study Population Characteristics

**Figure 1:**
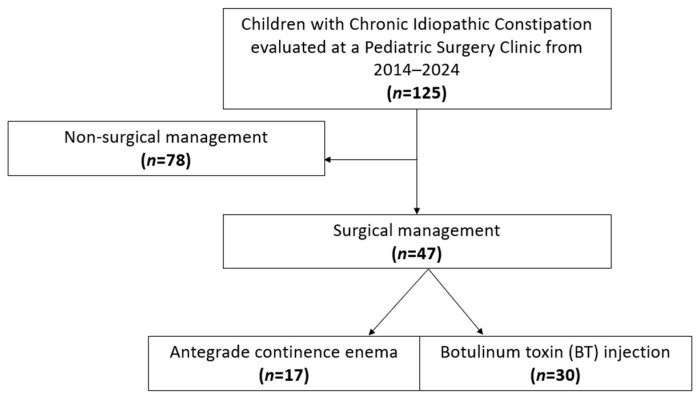
STROBE flowchart of study cohort.

A total of 125 patients met criteria for chronic idiopathic constipation, of whom 47 (37.6%) underwent surgery (Figure 1). Demographics, including sex and race/ethnicity, did not differ between surgical and non-surgical groups, nor did insurance type, and ADI percentile (Table 1). Surgical patients were significantly more likely to have a neurodiverse diagnosis (68.1% vs. 41.0%, *p* = 0.003). Constipation-related symptoms were common: abdominal pain was reported in 82% of patients, and abdominal distension in 67.2%, with no differences between surgical and nonsurgical groups. Surgical patients were more likely to present with encopresis (79.2% vs. 39.7%, *p* < 0.001). Twenty-one patients underwent lower gastrointestinal endoscopy for diagnostic evaluation, with 12 in the nonsurgical group and 9 in the surgical group. Only one patient, from the surgical group, had an abnormal finding of colonic redundancy. Laxative use was common (99.2%), with no significant difference in surgical and nonsurgical patients (100% vs. 98.7%, *p* = 1.0).

### 3.2. Characteristics of Surgical Patients

Among the 47 patients who underwent surgery, 17 (36.2%) received an ACE channel and 30 (63.8%) underwent BT injections alone. Ten ACE patients (58.8%) had a prior history of BT injection. The median interval between BT and ACE placement was 7.9 months (IQR 2.7–19.6 months). For this study, these patients were categorized within the ACE group, as they demonstrated no symptom improvement prior to ACE placement. Choice of treatment was determined through shared decision-making between patient and physician. The mean age at the time of surgery was 6.02 ± 2.60 years old. Within the ACE group, 9 patients (52.9%) had a Malone antegrade continence enema (MACE) and 8 (47.1%) underwent cecostomy. ACE reversal was performed in 8 patients (47.1%), with two cases due to treatment failure, from no improvement in abdominal distension, pain, and encopresis, and six following symptomatic improvement. The mean duration of ACE prior to reversal was 49.5 ± 22.6 months.

Of those treated with BT alone, most (90.0%) received a single injection, while 3 patients received two injections. In patients who received multiple BT injections, only the treatment response from the first BT treatment was included in our analysis. Compared with BT patients, ACE patients were significantly older at the time of presenting to a pediatric surgery clinic (7.72 ± 2.35 vs. 5.02 ± 1.93, *p* = 0.0001) and at surgery (9.10 ± 1.67 vs. 5.22 ± 1.85 years, *p* < 0.01). ACE patients also reported more frequent weekly bowel movements preoperatively (11.32 ± 10.76 vs. 4.37 ± 4.07 BM/wk, *p* = 0.003) and had a higher occurrence of encopresis prior to surgery (100% vs. 70.0%, *p* = 0.034). Demographics, neurodiverse disorder diagnosis, distance to hospital, ADI percentile, insurance, and daily laxative use did not differ significantly between groups at baseline (Table 2). Contrast enema was performed in 47.1% of ACE patients and 53.3% of BT patients, with abnormal findings in 0% and 12.5%, respectively (*p* = 0.536). Other preoperative symptoms were similar, including abdominal pain (94.1% vs. 86.7%, *p* = 0.761) and abdominal distension (52.9% vs. 70.0%, *p* = 0.393). Yearly CIC-related healthcare encounters before surgery were comparable between groups (5.33 ± 3.01 vs. 6.22 ± 5.17, *p* = 0.524).

### 3.3. Daily Oral Laxative Use in Surgical Patients

Oral laxative use was expectedly common among patients undergoing surgery, with 97.9% taking them at the time of surgery. Laxatives were categorized by mechanism of action (osmotic, stimulant, bulk-forming, stool softeners, secretagogues). Most patients (61.7%) were prescribed two or more classes, while 36.2% received a single class, and only 2.1% received none. Among specific laxative classes, osmotic agents were the most frequently prescribed (95.7%), followed by stimulants (56.5%). Fewer patients received secretagogues, including lubiprostone (4.3%) and linaclotide (2.2%), while none were prescribed stool softeners or bulk-forming agents. In the ACE group (n = 17), 58.8% of patients were prescribed two or more classes of laxatives, 35.3% received a single class, and one (5.9%) received none. Among the 16 patients prescribed laxatives at the time of surgery, osmotic agents were most frequently used in 93.8% of patients, followed by stimulants (56.3%) and secretagogues (25%). In the BT group (n = 30), 63.3% of patients were prescribed two or more classes and 36.7% a single class. Similarly, osmotic agents were the most common (96.7%), with stimulants (56.7%) and secretagogues (3.3%) prescribed less often.

**Figure 2:**
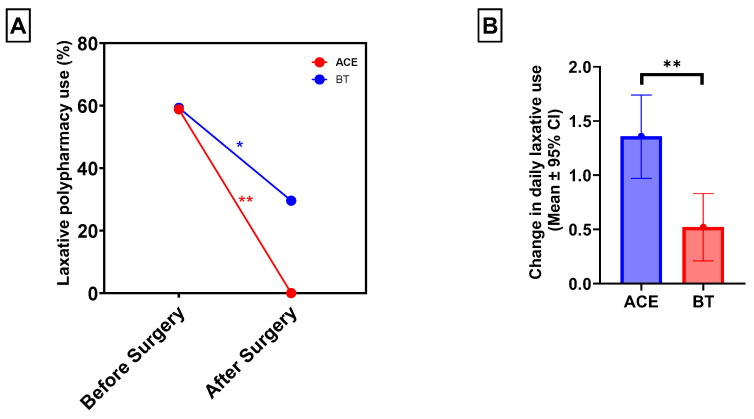
ACE and BT resulted in significantly reduced oral laxative polypharmacy use at one year postoperatively (**A**). ACE recipients had a significantly greater reduction in the number of daily oral laxative medications at one year postoperatively: 1.36 (95% CI: 0.97–1.75) vs. 0.52 (95% CI: 0.21–0.83) (**B**). * *p* < 0.05; ** *p* < 0.01.

For paired analysis before and after surgery, patients with missing documentation of daily oral laxative use at one year postoperatively were excluded. This resulted in a total of 44 patients out of the 47 who underwent surgery, including 17 in the ACE group and 27 in the BT group. Laxative use was significantly reduced in both groups at 1 year. Overall, 26 patients (59.1%) required two or more laxatives preoperatively, compared with only 8 patients (18.2%) postoperatively (McNemar’s χ^2^ = 14.0, *p* < 0.001). In the ACE group, 10 patients (58.8%) required polypharmacy (two or more laxatives) prior to surgery, whereas none required more than one laxative at 1 year (McNemar’s χ^2^ = 8.0, *p* = 0.004). In the BT group, polypharmacy decreased from 16 patients (59.3%) at baseline to 8 patients (29.6%) postoperatively (McNemar’s χ^2^ = 5.0, *p* = 0.030) (Figure 2A). While a higher percentage of patients in the ACE group showed improvement in polypharmacy compared to those who received BT, it was not statistically significant (58.8% vs. 29.6%, χ^2^ = 3.3, *p* = 0.068).

Among the 44 patients with 1-year follow-up data, the mean number of daily oral laxatives before surgery decreased significantly from 1.64 ± 0.67 to 0.77 ± 0.74 at 1 year postoperatively (*p* < 0.001). In the ACE group, daily laxative use decreased from 1.54 ± 0.79 preoperatively to 0.17 ± 0.39 at 1 year, *p* < 0.01). In the BT group, daily laxative use decreased from 1.70 ± 0.60 to 1.15 ± 0.66, 0.52; *p* = 0.002. After adjusting for age, sex, race/ethnicity, neurodiverse disorder, distance from hospital, and frequency of CIC-related visits, patients who underwent ACE experienced a significantly greater reduction in daily laxative use compared with those who received BT alone (adjusted mean difference: 0.94; 95% CI: −1.56 to −1.32; *p* = 0.005) (Figure 2B).

### 3.4. Chronic Constipation-Related Symptoms in Surgical Patients

We evaluated the impact of surgical intervention on constipation-related symptoms, including bowel movement frequency, encopresis, abdominal pain, and abdominal distension. Symptom data at 1 year were available for 43 patients (91.4%) among the surgical patients (n = 47). Bowel movement frequency did not significantly change (6.88 ± 7.88 BM/wk preoperatively vs. 7.99 ± 5.20 BM/wk at 1 year, *p* = 0.735). There were no differences in bowel movement frequency in ACE (11.53 ± 10.76 to 10.82 ± 5.38 BM/wk, *p* = 0.835) and BT (4.06 ± 3.95 to 5.58 ± 3.67 BM/wk, *p* = 0.336).

**Figure 3:**
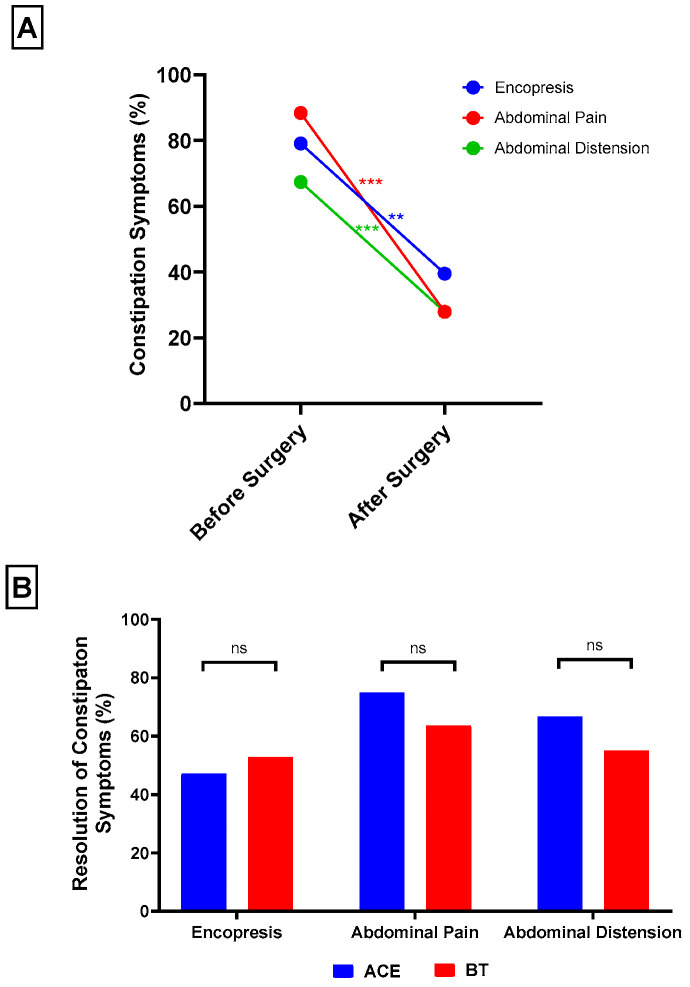
Overall, surgical patients experienced significant reduction in encopresis, abdominal pain, and abdominal distension at one year postoperatively (**A**). ACE and BT patients showed similar rates of resolution in encopresis, abdominal pain, and abdominal distension (**B**). ** *p* < 0.01; *** *p* < 0.001.

In contrast, encopresis improved markedly, decreasing from 34 patients (79.1%) preoperatively to 17 patients (39.5%) at 1 year (McNemar’s χ^2^ = 15.1, *p* = 0.001; Figure 3A). Abdominal pain was also significantly reduced, from 38 patients (88.4%) before surgery to 12 patients (27.9%) at 1 year (McNemar’s χ^2^ = 24.1, *p* < 0.001). Similarly, abdominal distension decreased from 29 patients (67.4%) to 12 patients (27.9%) (McNemar’s χ^2^ = 15.1, *p* < 0.001). Subgroup analyses demonstrated comparable resolution rates between ACE and BT groups for encopresis (47.1% vs. 52.9%, χ^2^ = 0.0, *p* = 1.00), abdominal pain (75.0% vs. 63.6%, χ^2^ = 0.2, *p* = 0.70), and abdominal distension (66.7% vs. 55.0%, χ^2^ = 0.03, *p* = 0.70; Figure 3B).

### 3.5. Chronic Idiopathic Constipation-Related Healthcare Visits in Surgical Patients

We evaluated healthcare utilization related to constipation before and after surgery. CIC-related visits were defined as encounters with any healthcare provider in which constipation was documented as a problem or in the encounter note. Among all surgical patients, the mean number of yearly outpatient CIC-related visits was similar before and after surgery (6.48 ± 6.02 vs. 6.83 ± 6.26, *p* = 0.70). Subgroup analyses revealed divergent trends: ACE patients had a significant increase in visits (5.33 ± 3.01 to 13.5 ± 5.09, *p* < 0.001), whereas BT patients had a significant decrease (6.52 ± 5.39 to 3.38 ± 2.58, *p* = 0.01). On univariate comparison, ACE patients had a mean increase of 8.2 visits per year, while BT recipients and a mean decrease of 3.2 visits (difference = −11.37 [95% CI: −14.7 to −8.1], *p* < 0.001). This difference remained significant in multivariate analysis adjusting for age, sex, race, neurodiversity, insurance type and distance from hospital (adjusted difference = −13.76 [95% CI: −18.85 to −8.67, *p* < 0.001]). No other covariates were significantly associated with change in visit frequency.

We also assessed constipation-related emergency department (ED) encounters. Overall, ED utilization did not differ significantly before and after surgery (0.55 ± 1.02 vs. 0.32 ± 0.84, *p* = 0.10). By surgery type, BT patients experienced a significant reduction (0.65 ± 1.02 to 0.15 ± 0.61, *p* = 0.003), while ACE patients had no significant change (0.53 ± 1.12 vs. 0.65 ± 1.11, *p* = 0.70).

Four patients (8.5%) experienced complications, including three in the ACE group (two with cecostomies and one with an MACE) and one in the BT group, within 30 days postoperatively. Two of the three patients in the ACE group with complications had a stoma surgical site infection, and the other experienced peri-stoma pain. The sole patient in the BT group had a complication of minor perianal bleeding.

## 4. Discussion

We found that a significant percentage of children (37.6%) referred to a surgeon received surgical intervention, including ACE channel creation and BT injection. Encopresis and neurodiversity were both associated with increased need for surgery. Our retrospective study demonstrates that both ACE and BT are effective surgical treatments for children with CIC. At one year postoperatively, daily laxative polypharmacy was reduced in 40.9% of patients, while encopresis, abdominal pain, and distension resolved in 39.6%, 60.5%, and 39.5% of patients, respectively. Notably, ACE recipients achieved a greater reduction in daily laxative use but experienced higher complication rates and more frequent CIC-related visits compared with patients treated with BT alone. These findings may help guide individualized clinical decision-making by identifying which patients are most likely to benefit from each intervention.

Pediatric CIC presents a challenge for surgeons, as evidence to guide surgical selection and predict response to treatment remains limited. In our study, treatment success was defined as reduced daily oral laxative use and symptom resolution at one year. ACE recipients achieved greater reductions in laxative use (58.5%) than BT recipients (29.6%), consistent with prior reports of effective long-term outcomes with ACE. Vriesman et al. reported that 73.9% and 52.2% of ACE patients discontinued laxatives at 6 and 8 months, respectively [20]. No prior studies have examined the effect of BT on oral laxative reduction. These differences in efficacy between ACE and BT may be in part explained by the distinct mechanisms of the two procedures. ACE channels provide direct access for antegrade delivery of irrigation with saline and the option of adding polyethylene glycol or stimulant-containing solutions, enabling mechanical emptying of the colon. BT injections, by contrast, act through temporary relaxation of the IAS, thereby reducing outlet obstruction and facilitating defecation [15,32,33]. However, because the pharmacologic effect of BT typically lasts only three to six months, its long-term impact on laxative reduction is variable. This transient effect of BT was observed in our cohort, where 10% of BT patients received more than one injection. The difference in efficacy between ACE and BT is also likely due to differences in patient selection between groups. In our cohort, ACE patients had more severe and longer duration of symptoms. Most (58.8%) of ACE patients had previously received BT injection and presumably had incomplete relief of symptoms, prompting escalation of surgical care. However, our observation of symptomatic improvement in BT recipients at 1 year is notable and suggests some patients with CIC may have long-lasting benefits from a single BT injection.

In addition to reducing laxative use, both ACE and BT improved constipation-related symptoms. Although bowel movement frequency did not significantly change from baseline to one year, other symptoms—including encopresis, abdominal pain, and distension—showed notable improvement. Encopresis, which is commonly due to overflow incontinence, is often a sign of severe constipation. It is particularly burdensome for children with CIC and is strongly associated with social stigma and reduced health-related quality of life [34,35]. In our cohort, encopresis was highly prevalent at baseline, especially among ACE recipients. One year after surgery, encopresis resolved with comparable improvement between ACE (47.1%) and BT (52.9%) recipients. Prior studies have also reported reductions in refractory encopresis following ACE, whereas the effect of BT has been more variable, with some reports showing improved stool frequency but limited impact on continence [36,37]. Abdominal pain and distension, reported by 88.4% in ACE and 67.4% in BT patients preoperatively, decreased to 27.9% at one year, with similar rates of relief between groups. Together, these findings highlight the effectiveness of both surgical interventions in not only reducing laxative use but also achieving meaningful symptom resolution.

Children with chronic constipation have higher healthcare utilization, including outpatient consultations, ED visits, and drug prescription costs, with overall expenditures estimated to be threefold greater than those of unaffected children [38]. Therefore, in addition to laxative use and symptom resolution, we examined healthcare utilization as an outcome of surgical treatment. In our cohort, the overall frequency of constipation-related encounters remained unchanged at one year compared with baseline. Subgroup analyses, however, revealed divergent trends: ACE recipients had significantly more outpatient visits at one year compared to baseline, whereas BT recipients had significantly fewer visits. ED encounters decreased among BT patients but remained unchanged in ACE patients. These findings suggest that although ACE effectively improves constipation-related symptoms and reduces laxative use, the need for maintenance care may contribute to greater postoperative healthcare utilization. ACE patients had a higher rate of complications than BT patients in our cohort, including peri-stoma pain and surgical site infection. Additionally, while nearly half of ACE patients ultimately underwent reversal, with the majority due to symptomatic improvement, some were due to treatment failure. Prior studies have reported mixed results on healthcare utilization after ACE, including decreased or unchanged outpatient visits but increased ED visits, largely related to device complications [34,39,40]. The increased need for healthcare utilization after ACE may also reflect greater disease severity in the ACE group, who likely required closer postoperative monitoring and medication adjustments, particularly within the first year.

This study has several strengths, including the direct comparison of two common surgical treatments for CIC, ACE and BT, and the assessment of laxative use, symptom resolution, and healthcare utilization over a ten-year period. However, several limitations should also be noted. First, the retrospective, single-center design and relatively small sample size limit the general application of our study. Similarly, nearly all surgical patients in this cohort presented with encopresis at baseline, which may not represent the broader CIC population. Next, few patients in our cohort underwent preoperative motility testing, such as anorectal or colonic manometry, which may help to guide and predict surgical treatment and response. Additionally, our analytic approach grouped patients who received ACE regardless of prior BT as ACE recipients because they demonstrated no symptom improvement prior to ACE placement. For BT analyses, outcomes were assessed after each patient’s first injection; these categorizations may have introduced bias. Another limitation of our study is that, given the relatively small size of the ACE cohort, we lacked the power to compare outcomes between ACE techniques (Malone appendicostomy vs. cecostomy). Finally, our study was limited to only postoperative outcomes in one year, and data accuracy was dependent on of doctors’ notes in the electronic medical record. Future prospective multicenter studies comparing the effects of surgical options on relieving constipation symptoms and healthcare costs are necessary, especially in children with neurodiverse diagnoses, who have consistently shown higher rates of constipation that are refractory to medical therapies [30,41].

## 5. Conclusions

In summary, both ACE and BT injections were effective surgical options for pediatric chronic idiopathic constipation. ACE resulted in greater reduction in laxative burden and resolution of constipation symptoms at the cost of increased postoperative healthcare utilization. These findings highlight the importance of an individualized approach in selecting the most appropriate management and examining opportunities to optimize postoperative care in this population.

## Figures and Tables

**Table 1:** Baseline characteristics of pediatric patients with Chronic Idiopathic Constipation.

	Non-Surgery (*n* = 78)	Surgery (*n* = 47)	*p*-Value
Male	42 (53.8%)	27 (57.4%)	0.695
Race			0.635
White	61 (78.2%)	37 (78.7%)	
Black	9 (11.5%)	7 (14.9%)	
American Indian and Alaska Native	0 (0%)	1 (2.1%)	
Other	8 (10.3%)	2 (4.3%)	
Ethnicity			0.995
Hispanic	5 (6.4%)	3 (6.4%)	
Non-Hispanic	73 (93.6%)	44 (93.6%)	
Insurance			0.756
Private	25 (32.1%)	18 (38.3%)	
Government	52 (66.67%)	29 (61.7%)	
None	1 (1.3%)	0 (0%)	
Diagnosis of neurodiverse disorder	32 (41.0%)	32 (68.1%)	0.003
Home-to-hospital distance (mean ± SD)	68.3 ± 39.0	86.9 ± 71.6	0.105
ADI percentile (mean ± SD)	52.8 ± 20.2	51.8 ± 21.6	0.790
Lower endoscopy	12 (15.4%)	9 (19.1%)	0.623
Abdominal pain	61 (78.2%)	42 (89.4%)	0.113
Abdominal distension	54 (69.2%)	30 (63.8%)	0.533
Encopresis	31 (39.7%)	38 (79.2%)	<0.001
Laxative use	77 (98.7%)	47 (100%)	1.000

**Table 2:** Baseline characteristics of surgical pediatric patients with Chronic Idiopathic Constipation.

	Antegrade Continence Enemas (*n* = 17)	Botulinum Toxin Only (*n* = 30)	*p*-Value
Male	11 (64.7%)	16 (53.3%)	0.652
Age at time of surgery	9.10 ± 1.67	5.22 ± 1.85	<0.001
Age at surgery consult	7.72 ± 2.35	5.02 ± 1.93	<0.001
Race			0.829
White	13 (76.5%)	24 (80%)	
Black	3 (17.6%)	4 (13.4%)	
American Indian and Alaska Native	0 (0%)	1 (3.3%)	
Other	1 (5.9%)	1 (3.3%)	
Ethnicity			0.467
Hispanic	0 (0%)	3 (10%)	
Non-Hispanic	17 (100%)	27 (90%)	
Insurance			0.346
Private	5 (29.4%)	13 (43.3%)	
Government	12 (70.6%)	17 (56.7%)	
None	0 (0%)	0 (0%)	
Diagnosis of neurodiverse disorder	14 (82.4%)	18 (60%)	0.210
Home-to-hospital distance (mean ± SD)	90.59 ± 74.23	84.87 ± 71.21	0.796
ADI percentile (mean ± SD)	54.2 ± 21.2	50.5 ± 22.1	0.590
Contrast enema	8 (47.1%)	16 (53.3%)	0.679
Abnormal contrast enema	0 (0.0%)	2 (12.5%)	0.536
Abdominal pain before surgery	16 (94.1%)	26 (86.7%)	0.761
Abdominal distension before surgery	9 (52.9%)	21 (70.0%)	0.393
Encopresis before surgery	17 (100%)	21 (70.0%)	0.034
Pre-operative BM/week (mean ± SD)	11.32 ± 10.76	4.37 ± 4.07	0.003
Pre-operative laxatives/day (mean ± SD)	1.54 ± 0.79	1.70 ± 0.60	0.424
Pre-operative CIC-related healthcare encounters/year (mean ± SD)	5.33 ± 3.01	6.22 ± 5.17	0.524

## Data Availability

In accordance with data sharing guidelines and to facilitate transparency in research, the data upon which this study was based is available upon request.

## Abbreviations

The following abbreviations are used in this manuscript:

CIC	Chronic Idiopathic Constipation
BT	Botulinum Toxin
ACE	Antegrade Continence Enema
MACE	Malone Antegrade Continence Enema
IAS	Internal Anal Sphincter
GI	Gastrointestinal
BM/wk	Bowel movement per week
ASD	Autism spectrum disorder
IDD	Intellectual and developmental disability
ADHD	Attention-deficit/hyperactivity
SD	Standard deviation
